# Structured skin care for a patient with toxic epidermal necrolysis caused by anlotinib: Case report

**DOI:** 10.1097/MD.0000000000044955

**Published:** 2025-09-26

**Authors:** Jinjin Shao, Liqiu Sun, Yan Ma, Meiqi Yao

**Affiliations:** a Nursing Department, Sir Run Run Shaw Hospital, Zhejiang University School of Medicine, Hangzhou, China; b The Second Affiliated Hospital of Zhejiang University School of Medicine, Hangzhou, China.

**Keywords:** pancreatic cancer, structured skin care, toxic epidermal necrolysis

## Abstract

**Rationale:**

Toxic epidermal necrolysis is a life-threatening disease. Over 95% of toxic epidermal necrolysis cases are caused by drugs, presenting as hemorrhagic erythema, blisters, and extensive epidermal detachment on the skin and mucous membranes. It is usually accompanied by multiple system damage. According to the size of the exfoliated area, toxic epidermal necrolysis is classified as severe (exfoliation area >30% of body surface area).

**Patient concerns:**

A 78-year-old female patient was admitted to our Department of Medical Oncology due to “after pancreatic cancer surgery for >9 months, discovery of liver metastasis for >1 months, multiple oral ulcers accompanied by multiple rashes throughout the body, with large red exfoliative maculopapule on the hands and feet for 5 days.”

**Diagnoses:**

Postoperative status of pancreatic cancer, liver metastasis, and toxic epidermal necrolysis.

**Interventions:**

After discontinuing anlotinib, the skin symptoms did not subside. Therefore, structured skin care was started.

**Outcomes:**

The skin symptoms was resolved after starting structured skin care.

**Lessons:**

In this case, there was only 1 patient, and the nursing care was relatively limited. The effect needs further verification.

## 1. Introduction

Anlotinib is a novel multi-target receptor tyrosine kinase inhibitor. It can effectively inhibit the activity of platelet-derived growth factor receptor, vascular endothelial growth factor receptor, fibroblast growth factor receptor, mast/stemcell growth factor receptor and other kinases.^[1,2]^ Toxic epidermal necrolysis is a life-threatening disease. Over 95% of toxic epidermal necrolysis cases are caused by drugs, presenting as hemorrhagic erythema, blisters, and extensive epidermal detachment on the skin and mucous membranes. It is usually accompanied by multiple system damage. According to the size of the exfoliated area, toxic epidermal necrolysis is classified as severe (exfoliation area >30% of body surface area).^[3,4]^ Shao et al^[5]^ reported a real study on the treatment of advanced malignant tumors by anlotinib. The incidence of adverse skin reactions caused by anlotinib was 29.31%, among which 3.44% were toxic epidermal necrolysis. The treatment and nursing plan for serious skin adverse reactions caused by targeted drugs such as anlotinib are still under constant exploration. The difficulties in nursing are mainly reflected in the lack of sound management monitoring and practical workflow which makes it impossible for nurses to take effective measures in time. In December 2023, our hospital successfully treated a pancreatic cancer patient with toxic epidermal necrolysis combined with fungal diarrhea and stomatitis caused by the use of anlotinib. After overall fine nursing and scientific implementation of early rehabilitation training, the patient’s hand, foot and mouth wounds were completely healed, the perianal skin was intact, and the diarrhea was cured. Oncology clinic follow-up after discharge. The nursing experience is reported as follows.

## 2. Case presentation

A 78-year-old female patient, after pancreatic cancer surgery for >9 months, discovery of liver metastasis for >1 months, multiple oral ulcers accompanied by multiple rashes throughout the body, with large red exfoliative maculopapule on the hands and feet for 5 days. On December 7, 2023, the patient received targeted therapy combined with chemotherapy for antitumor treatment using “anlotinib hydrochloride capsules + tegafur, gimeracil and oteracil potassium capsules” orally, discharged after treatment. Five days after discharge, the patient developed multiple oral ulcers that caused difficulty in eating, accompanied by multiple rashes throughout the body, a slight feeling of dull pain in the hepatic region, fever but no chills, abdominal distension, and diarrhea. For these symptoms, the patient was readmitted and received a physical examination with a body temperature of 38.5°C, yellow loose and soft stool for 10 times in 24 hours, 10% of *Candida albicans* in stool culture, incontinence-associated dermatitis with skin lesions of the perianal skin, white blood cell count of 1.3 × 10^9^/L, hypersensitive C-reaction protein of 60.1 mg/L, albumin of 29.3 g/L, electrolyte disturbance. Purplish red exfoliative maculopapule on the neck, upper chest, waist and back, thighs, hands and feet with pain, erythema and ulceration of lips with pain, visible purulent discharge, inability to eat orally, weight loss of 2 kg, severely reduced self-care ability, activities of daily living (ADL) grade III, and declined sleep quality due to pain. After symptomatic treatment such as anti-infection, promoting mucosal repair, and early rehabilitation, the therapeutic effect was evaluated. Skin improvement evaluation: significant improvement (herpes, ulceration, pruritus, erythema, pus, and other symptoms were significantly improved, and the improvement range was ≥90%); Improvement (improvement of the above skin symptoms, and 90% > improvement margin ≥ 40%); Ineffective (no significant improvement or deterioration of skin symptoms), the patient’s skin improvement was significantly improved. Pain degree: nutritional risk screening (NRS) scoring method was used to evaluate the pain degree, 0 was classified as no pain; ≤3 is classified as mild pain, tolerable, psychological care is the main, 4 to 6 is classified as moderate pain and affect sleep, need drug intervention, ≥7 is classified as severe pain, unbearable. The patient was admitted with an NRS score of 4 to 5, after 1 week of treatment, the NRS score was 2 to 3, and after 2 weeks, the NRS score was about 1, and finally the pain completely disappeared. Self-care ability: ADL score was used to evaluate self-care ability, and the patient reached ADL level Ⅰ. Satisfaction: The score includes the professionalism of nursing work, patients’ subjective feelings and comfort, diet, ward environment, etc. The higher the score, the better the patient satisfaction, and the score of patient satisfaction is 100.

## 3. Discussion

The concept of structured skin care was first proposed by foreign medical personnel and mainly applied to incontinence-associated dermatitis. The relevant skin care guidelines written and issued by International Wound Management Professional Organizations and Beijing Nursing Society both indicate the important guiding significance of structured skin care programs for skin management.^[6]^ Structured skin care: By assessing the whole body and local skin conditions of patients, according to the specific conditions of patients, under the framework of problems, with the goal of solving problems, the whole process of intervention is implemented in accordance with certain structural design in steps and contents, so as to maintain or promote the skin health of patients.^[7]^

### 3.1. Set up a structured skin care management team

The director of the department is the group leader, the head nurse is the deputy group leader, and there are 6 team members and 5 specialist nurses. The management team will carry out full treatment, nursing and rehabilitation for patients.

### 3.2. Develop a structured skin care proposal

Structured management mode was applied, high-quality references were retrieved, structured skin care program was determined, and finally structured skin care program was developed. There were 4 projects in total. The content reliability and validity test was conducted by the hospital wound and stomatology incontinence specialist nurse, the hospital dermatology experts were invited to evaluate the content validity, and the modification and improvement were discussed in the form of seminars.

### 3.3. Guide nursing implementation based on skin repair mechanism

#### 3.3.1. Early dynamic skin assessment

Skin assessment is the first step in skin care, providing an opportunity for early identification and treatment of abnormal skin conditions. Within 2 hours after the patient entered the ward, CTC AE5.0 criteria was used for evaluation,^[8]^ and the evaluation evidence level was level 5, level A recommended, which was more reliable. The patient belonged to grade G3, and the evaluation frequency was assessed every shift to observe whether erythema, ulceration, exudation, numbness and other conditions occurred, and it was evaluated at any time when there were changes.

#### 3.3.2. Discontinuation of medication

The patient’s skin adverse reactions had reached a serious level, including G3 grade combined fungal diarrhea and stomatitis, which seriously affected his quality of life, severely decreased physiological ability, accompanied by pain, and severe drug side effects. The patient was treated according to grade 3, and anlotinib was taken orally.^[9]^

#### 3.3.3. Clean the skin

While the patient is in bed, the area with skin symptoms should be cleaned regularly, and care should be taken to avoid any scratching or scratching. In addition, use soft disposable wipes or towels, dip in a small amount of weakly acidic cleaning solution or saline, do not drip as appropriate, wipe slowly, do not apply too much force.

#### 3.3.4. Skin protection

Use unscented bath oils and warm moisturizing bath soaps; avoid cold and dry climate to go out; avoid sunlight; use nonalcoholic skin care products, apply urea cream evenly on hands and feet, spray-oral mucosa, ostomy powder, liquid protective film to protect perianal skin.

### 3.4. Skin repair

#### 3.4.1. Inflammatory stage

The inflammatory stage is divided into 2 processes: vascular response (hemostasis) and cellular response (inflammation), which are mainly manifested as redness, swelling, pain, and inflammatory exudation. The epidermal injury usually has no bleeding or only a small amount of bleeding, and can last up to 6 days. In order to prevent and control infection, the skin treatment at this stage should be isolated in a single room. Blood culture, throat swab and stool culture should be performed to determine the nature of infection before symptomatic treatment. In case of suppurative granuloma, nail avulsion, electrolysis, and silver nitrate are recommended to remove excessive granulomatous tissue. If the skin ruptures, it is recommended to use diluted vinegar, 3% hydrochloric acid or boric acid solution, hydrogen peroxide solution for cleaning, sterile transparent dressing, local or systemic use of hormones and iodine solution treatment.^[10]^

#### 3.4.2. Stages of organization formation

In the tissue formation stage, the damaged tissue of the epidermis and dermis is formed as a reepithelialization process, also known asepithelial replacement, that is, the new epithelium replaces the damaged epithelial tissue. Under normal circumstances, the reepithelialization process can be completed 7 to 10 days after the injury. In this stage, the wet healing theory is followed to increase wound permeability, maintain wound moistness,^[11]^ promote healing and relieve pain. According to the summary of evidence of the prevention and management of skin adverse reactions in patients with tumor targeted therapy by Lisa Hu et al^[9]^ dry gel, Kangfuxin wet compress, honeysuckle solution, aloe veragel, antibacterial moisturizer, hydrophilic dressing, and preservative treatment are recommended.

#### 3.4.3. Stage of tissue shaping

The tissue shaping stage is also known as the mature stage, in which the regenerated epithelium gradually returns to normal feeling, elasticity, tensile strength and tensile strength, and the color is gradually close to the surrounding tissue.^[12,13]^ This stage may take about 6 months, and continuity care is the main focus. In this stage, avoid irritation, protect the skin, promote shaping, wear loose cotton clothes, relax properly, and avoid friction. Change your bedding regularly, keep your bed clean and dry, and take care of sun protection.

The patient was in the tissue shaping stage after 21 days with dry, healed skin.

### 3.5. Implementation of early sequential enteral nutrition^[14]^

#### 3.5.1. Establish a nutrition management team

The patient’s albumin was 29.3 g/L, potassium was 3.94 mmol/L, sodium was 136 mmol/L, body weight was reduced by 2 kg, BMI 16.5, lip erythema and erosion ulcer with pain, purulent secretions could be seen, and eating through mouth was difficult, all indicators suggested that the patient had nutritional problems. A nutrition management team should be established to develop an individualized whole-process nutrition management plan for patients. A nutrition management team was formed that included a chief medical oncologist, a pharmacist, a dietitian and a nutrition specialist nurse. Among them, the nutrition specialist nurses bear the main responsibility of comprehensive evaluation of the nutritional status of patients, and jointly formulate and dynamically adjust the nutrition management plan with other team members.

#### 3.5.2. Pay attention to nutritional risk screening and nutritional assessment

To ensure that patients receive personalized nutrition management programs, early nutritional risk screening and nutritional assessment are essential foundational work.^[15]^ The nutrition specialist nurse, together with other members, comprehensively assessed the patient’s relevant medical history, blood biochemical examination, diet, and NRS 4 score of the patient for NRS 2002 to determine the presence of malnutrition in the patient and establish a nutrition management file.

#### 3.5.3. Initiating early sequential enteral nutrition

According to the standard of 80 to 130 kJ/kg daily supply of calories, the first 3 days with short peptide-based enteral nutrition preparations such as baipuli (SP), orally 50 to 100 mL/time, once every 4 hours, the dosage of 500 mL on the first day, if no adverse reactions such as diarrhea, abdominal distension, nausea and vomiting, gradually increased to 150 to 180 mL/time. Then increase 500 mL/d until reaching the full dose of 1500 to 2000 mL/d.^[16]^ From the 4th day, the whole protein type enteral nutrition preparation such as full vegetarian, the oral method and amount of the same as baipuli (SP)l, continued to be given for 11 days. When the temperature of nutrient solution is controlled at 15 to 25°C, cool diet is more effective than warm diet for patients’ tolerance and appetite improvement, alleviating oral pain, improving comfort and nursing satisfaction.^[17]^

#### 3.5.4. Develop individualized parenteral nutrition program

In the case that enteral nutrition has not yet begun to implement or enteral nutrition is insufficient, parenteral nutrition should be implemented in time to maintain the nutritional needs of the body. Specialist nurses, dietitians, pharmacists and other relevant personnel jointly formulate an individualized parenteral nutrition plan according to the parenteral nutrition guidelines,^[15]^ including: First, daily energy intake should be 60 to 80 kJ/kg as the starting point, and the target value should be 200 to 300 kJ/kg within 3 to 4 days. Second, when the patient’s vital signs and internal environment were stable, periodic parenteral nutrition treatment was given, that is, the parenteral nutrition infusion time was shortened by 1 to 2 hours every 2 days until the daily infusion time was 12 to 18 hours, in order to promote nutrient absorption and reduce parenteral nutrition-related complications. Third, blood glucose monitoring was performed every 4 hours, and the monitoring was stopped when the blood glucose was <7.8 mmol/L for 24 hours. Fourth, blood biochemical test was performed once a week, and parenteral nutrition program was timely adjusted according to albumin concentration, liver and kidney function, total protein and other indicators.

With the above measures, the patient’s albumin increased to 33.5 g/L, electrolytes were normal, and body weight remained stable.

### 3.6. Step rehabilitation nursing

#### 3.6.1. Fall prevention

In literature studies, falls occur in about 18% of patients with toxic epidermal necrolysis,^[10,18]^ which may be caused by hand and foot numbness and numbness, resulting in unsteady gait and body balance dysfunction. Fall risk was assessed with Morse Fall Risk Rating Scale. Our department recorded education videos on fall prevention, which were watched by patients on tablet computers during hospitalization, and then emphasized verbally by nurses. Finally, the Teach-back health education model was adopted for evaluation.

#### 3.6.2. Stage 1: Bed activities

Due to numbness and pain in the hands and feet, patients form kinetophobia, fear of activity, and mainly stay in bed, while exercise can alleviate the discomfort of numbness and pain in the hands and feet caused by peripheral neuropathy induced by targeted drugs. It is considered appropriate for cancer patients to do aerobic exercise, such as jogging and cycling, which requires hands and feet to touch the ground. In order to achieve the purpose of exercise and avoid prolonged pressure and friction on hands and feet, the department downloaded the exercise video of the supine cycling exercise method, which can completely avoid the contact between hands and feet and any objects, strengthen the exercise of hospitalized patients and emphasize the specific exercise time and details. For example, it is recommended to lay a hard board when exercising.^[10]^

#### 3.6.3. Stage 2: Bed side activities

Assist the patient to sit up, let the patient sit on the edge of the bed, feet touch the ground, initial feeling, time 5 to 10 minutes, and then exercise the balance of standing and walking, and then according to their tolerance level with the support of family members gradually bedside walking exercise, time control within 5 minutes.

#### 3.6.4. Stage 3: Inpatient activities

The master degree of rehabilitation exercise was assessed according to the standing and balance training of the patients, and gradually transferred to activities in the ward or using a walker. The activity time was 10 to 20 minutes.

#### 3.6.5. Stage 4: Continuous rehabilitation nursing

After discharge, once a week of follow-up and every 2 weeks of hospital treatment to understand the rehabilitation exercise of patients, and give personalized guidance, according to the specific conditions of patients to guide them to do medical training exercises to promote the recovery of limb function. The intervention lasted for 1 month.

Through the early rehabilitation activity program from local activities to whole body exercise, from bed exercise, bedside exercise, inpatient exercise to continuous rehabilitation nursing, follow the principle of gradual progress, improve patients’ self-care ability, and finally reach ADL grade I.

In conclusion this case suggests that CTC AE 3G, stopping the medication is the primary measure. However, the treatment of toxic epidermal necrolysis requires professional and structured skin care to achieve rapid skin repair, alleviate complications, improve patient comfort and satisfaction. However, in this case, there was only 1 patient, and the nursing care was relatively limited. The effect needs further verification.

## Author contributions

**Conceptualization:** Liqiu Sun.

**Formal analysis:** Jinjin Shao.

**Funding acquisition:** Jinjin Shao, Liqiu Sun.

**Investigation:** Liqiu Sun.

**Resources:** Jinjin Shao, Yan Ma.

**Software:** Yan Ma, Meiqi Yao.

**Supervision:** Yan Ma, Meiqi Yao.
